# Development and Validation of a Deep Learning Model to Reduce the Interference of Rectal Artifacts in MRI-based Prostate Cancer Diagnosis

**DOI:** 10.1148/ryai.230362

**Published:** 2024-03-06

**Authors:** Lei Hu, Xiangyu Guo, Dawei Zhou, Zhen Wang, Lisong Dai, Liang Li, Ying Li, Tian Zhang, Haining Long, Chengxin Yu, Zhen-wei Shi, Chu Han, Cheng Lu, Jungong Zhao, Yuehua Li, Yunfei Zha, Zaiyi Liu

**Affiliations:** From the Guangdong Cardiovascular Institute, Guangdong Provincial People’s Hospital, Guangdong Academy of Sciences, Guangzhou, China (L.H.); Department of Radiology, Guangdong Provincial People’s Hospital (Guangdong Academy of Medical Sciences), Southern Medical University, No. 106 Zhongshan Er Road, Guangzhou 510080, China (L.H., Z.W.S., C.H., C.L., Z.L.); Guangdong Provincial Key Laboratory of Artificial Intelligence in Medical Image Analysis and Application, Guangzhou, China (L.H., Z.W.S., C.H., C.L., Z.L.); Department of TPS Algorithm, Xi’an OUR United Corporation, Xi’an, China (X.G.); State Key Laboratory of Integrated Services Networks, School of Telecommunications Engineering, Xidian University, Xi’an, China (D.Z.); Department of Radiology, Yichang Central People’s Hospital Affiliated to the First Clinical Medical College of Three Gorges University, Yichang, China (Z.W., C.Y.); Institute of Diagnostic and Interventional Radiology, Shanghai Sixth People’s Hospital Affiliated to Shanghai Jiao Tong University School of Medicine, Shanghai, China (L.D., H.L., J.Z., Yuehua Li); and Department of Radiology, Renmin Hospital of Wuhan University, Wuhan, China (L.L., Ying Li, T.Z., Y.Z.).

**Keywords:** MR–Diffusion-weighted Imaging, Urinary, Prostate, Comparative Studies, Diagnosis, Transfer Learning

## Abstract

**Purpose:**

To develop an MRI-based model for clinically significant prostate cancer (csPCa) diagnosis that can resist rectal artifact interference.

**Materials and Methods:**

This retrospective study included 2203 male patients with prostate lesions who underwent biparametric MRI and biopsy between January 2019 and June 2023. Targeted adversarial training with proprietary adversarial samples (TPAS) strategy was proposed to enhance model resistance against rectal artifacts. The automated csPCa diagnostic models trained with and without TPAS were compared using multicenter validation datasets. The impact of rectal artifacts on the diagnostic performance of each model at the patient and lesion levels was compared using the area under the receiver operating characteristic curve (AUC) and the area under the precision-recall curve (AUPRC). The AUC between models was compared using the DeLong test, and the AUPRC was compared using the bootstrap method.

**Results:**

The TPAS model exhibited diagnostic performance improvements of 6% at the patient level (AUC: 0.87 vs 0.81, *P* < .001) and 7% at the lesion level (AUPRC: 0.84 vs 0.77, *P* = .007) compared with the control model. The TPAS model demonstrated less performance decline in the presence of rectal artifact–pattern adversarial noise than the control model (ΔAUC: −17% vs −19%, ΔAUPRC: −18% vs −21%). The TPAS model performed better than the control model in patients with moderate (AUC: 0.79 vs 0.73, AUPRC: 0.68 vs 0.61) and severe (AUC: 0.75 vs 0.57, AUPRC: 0.69 vs 0.59) artifacts.

**Conclusion:**

This study demonstrates that the TPAS model can reduce rectal artifact interference in MRI-based csPCa diagnosis, thereby improving its performance in clinical applications.

**Keywords:** MR–Diffusion-weighted Imaging, Urinary, Prostate, Comparative Studies, Diagnosis, Transfer Learning

Clinical trial registration no. ChiCTR23000069832

*Supplemental material is available for this article.*

Published under a CC BY 4.0 license.

## Graphical abstract

**Figure:**
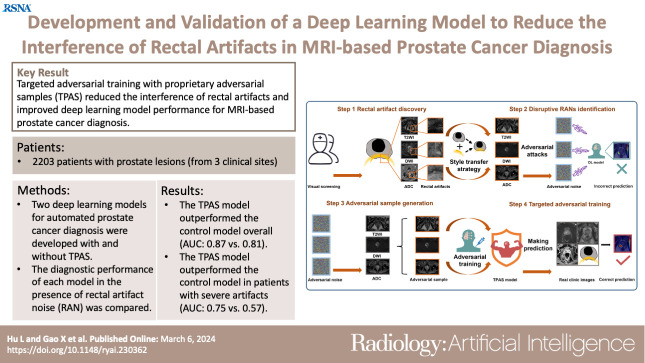

**Summary**

Targeted adversarial training with proprietary adversarial samples reduced the interference of rectal artifacts and improved deep learning model performance for MRI-based prostate cancer diagnosis on a multicenter dataset.

**Key Points**

■ The targeted adversarial training with proprietary adversarial samples model outperformed the control model for MRI-based prostate cancer diagnosis in a multicenter study including 2203 patients (area under the receiver operating characteristic curve [AUC]: 0.87 vs 0.81, *P* < .001; area under the precision-recall curve [AUPRC]: 0.84 vs 0.77, *P* = .007).
■ The model significantly outperformed the control model in patients with severe rectal artifacts (AUC: 0.75 vs 0.57, *P* < .001; AUPRC: 0.69 vs 0.59, *P* = .002).

## Introduction

In recent years, various deep learning (DL) models based on MRI have shown promising results in the diagnosis of prostate cancer (PCa). However, most of these models, including U.S. Food and Drug Administration–certified commercial software, exhibit accuracy declines in real clinical scenarios, ranging from 3% to 22% (1,2). The limited training data and complex and varied image quality pose substantial obstacles for these DL models to achieve similar and stable performances in different datasets. Consequently, these DL models do not have full clinical trust.

Prostate MR images suffer from various artifacts, particularly rectal artifacts, which are common (3–6). A previous study highlighted the negative effect of rectal artifacts on the performance of DL models for PCa diagnosis. Rectal susceptibility artifacts demonstrate independent risk factors associated with both false-positive (odds ratio, 1.12 [95% CI: 1.05, 1.21]) and false-negative (odds ratio, 5.46 [95% CI: 2.77, 10.96]) detections (2). Mitigating rectal artifacts is crucial for improving DL performance in practical clinical applications. However, mitigating these artifacts poses technical challenges. Although current clinical strategies, such as bowel preparation before MRI, and advanced imaging technologies can partially mitigate the impact of these artifacts, complete elimination of their effect remains difficult (7,8). Additionally, subtle rectal artifact noise (RAN) is challenging to identify visually but can still affect DL models. Deep neural networks are vulnerable to specific types of small noise (9,10), and previous research demonstrated that certain RAN can disrupt the functioning of PCa diagnostic models with different structures that previously performed well (11,12). However, this issue has not received sufficient attention. Most previous prostate MRI-based DL studies typically excluded images with artifacts from training sets simply by visual inspection (1,4), and none of the DL models developed specific strategies to address subtle noise interference. Consequently, these challenges remain unresolved.

In clinical practice, vaccination is commonly used to boost immune response against specific pathogens. Inspired by this, we propose a DL training strategy called *targeted adversarial training with proprietary adversarial samples* (TPAS), which uses an inoculation strategy to enhance the resistance of the DL model to rectal artifacts. In this study, we developed an automated model for clinically significant PCa (csPCa) diagnosis. We evaluated the impact of the interference of visible rectal artifacts and invisible RAN on the DL model. The performances of the model with and without TPAS were assessed and compared using a multicenter validation dataset.

## Materials and Methods

This retrospective multicenter study was approved by the local ethics committee, and the requirement for informed consent was waived due to the retrospective and anonymous analysis of the data (approval no. KY2023–146–01). The study is registered at *www.chictr.org.cn* (registration no. ChiCTR2300069832).

### Patients

The annotated public dataset Prostate Imaging: Cancer AI (version 1.1) was used for model training and internal testing. It comprises 1500 anonymized prostate MRI scans in 1476 patients collected from three centers (Radboud University Medical Center, University Medical Center Groningen, and Ziekenhuisgroep Twente). More detailed information on this dataset is provided in Table S1. This dataset can be accessed at *https://zenodo.org/record/6667655* (13).

**Figure 1:**
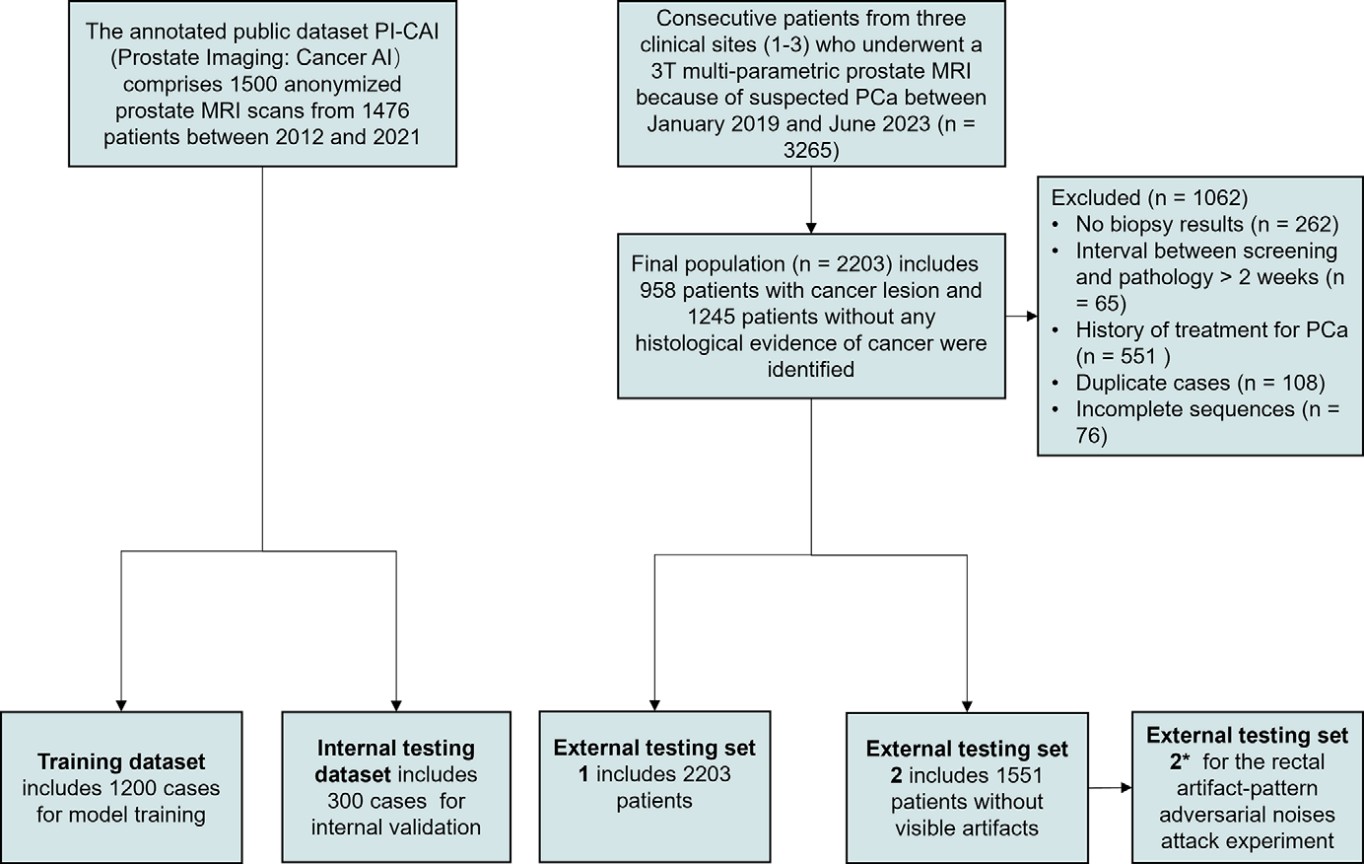
Diagram for inclusion of patients into the study. The PI-CAI public dataset was randomly divided in a 4:1 ratio for model training and internal testing. External testing set 1 assessed model performance in a clinical setting. External testing set 2 contained 1551 cases without visible artifacts. External testing set 2*, sharing the same patients as set 2, had subtle, invisible rectal artifact–like noises added to the images. External testing sets 2 and 2* were used to conduct a rectal artifact–pattern adversarial noise attack experiment to assess rectal artifact noise interference on the DL model. DL = deep learning, PCa = prostate cancer, PI-CAI = Prostate Imaging: Cancer AI.

For external testing of the model, we initially included 3265 patients who underwent preoperative MRI examinations and subsequent MRI fusion US-guided biopsies for suspected PCa from three different clinical sites (referred to as Cs 1–3) between January 2019 and June 2023. The inclusion criteria were as follows: *(a)* imaging performed using a 3-T MRI system and *(b)* MRI fusion US-guided targeted biopsies were performed for MRI-suspicious lesions, followed by systematic biopsies. We excluded 262 patients from the analysis because of lack of biopsy results; 65 patients because of a longer than 2-week interval between screening and pathology; 551 patients because of a history of previous treatment for csPCa, such as antihormonal therapy, radiation therapy, focal therapy, cryotherapy, or surgery before MRI; 108 patients because of duplicate cases; and 76 patients because of incomplete sequences (Fig 1).

Data from 2203 consecutive patients from Cs 1–3 were finally used as external testing set 1 to evaluate the performance of the DL model in a real clinical setting. Among these 2203 patients, 29.6% (652 of 2203) had rectal artifacts with varying severity (232, 222, and 198 patients had mild, moderate, and severe artifacts, respectively). Patients without visible artifacts (1551 of 2203) formed external testing set 2. To assess the impact of RAN interference on the DL model, subtle, invisible rectal artifact–like noises were intentionally introduced into external testing set 2 (referred to as external testing set 2*) to conduct a rectal artifact–pattern adversarial noise attack experiment.

Previous studies have evaluated 899 patients in the external testing sets (2,14–16). These previous studies explored various aspects of DL in medical imaging. This includes evaluating whether generating synthetic high-*b*-value diffusion-weighted imaging (DWI) (14) and synthetic apparent diffusion coefficient (ADC) studies (15) using DL methods could enhance the diagnostic efficiency for csPCa diagnosis, comparing the diagnostic differences in csPCa classification using radiomics models constructed from different DWI techniques (16) and assessing potential confounding risk factors that might interfere with DL-based computer-assisted diagnosis of csPCa (2). The current study assesses the use of our proposed TPAS strategy to enhance the DL model’s resilience against the identified risk factor, specifically, rectal artifacts.

### MRI Examination

The imaging protocol of the training set included standardized precautionary measures, such as minimizing time gaps between acquisitions, administering antispasmodic agents to reduce bowel motility, and using rectal catheters to minimize distension. A clinical biparametric MRI examination including T2-weighted imaging, high-*b*-value DWI, and ADC was performed using 1.5-T or 3-T MRI scanners (Siemens Healthineers or Philips Medical Systems) equipped with surface coils (17).

In the external testing sets, patients from Cs 1 and 2 followed an 8-hour clear fluid diet and emptied their rectum before undergoing MRI. Patients from Cs 3 did not undergo rectal preparation before MRI. Imaging was performed using one of three 3-T MRI scanners with a standard multichannel body coil. Similarly, for external testing datasets, a biparametric MRI protocol that included T2-weighted imaging, DWI, and ADC was used in all datasets. The specific parameters of each MRI sequence are presented in Table S2.

### Histopathologic Matching and Lesion Annotation

Uropathologists conducted histopathologic analyses in accordance with the International Society of Urological Pathology standards and obtained the Gleason score from the patient’s pathologic report. Lesions that were found to be benign or Gleason grade group 1 were grouped as clinically insignificant diseases. Lesions within Gleason grade groups 2 to 5 were classified as csPCa (18).

The lesion locations were reported by or under the supervision of one of the three expert radiologists with more than 20 years of experience in prostate MRI. The findings were confirmed with an MRI-guided biopsy. All patients underwent systematic biopsies, two cores from each of the 12 anatomic sectors. Further, two cores were biopsied per target lesion. Histopathologic analysis was correlated with MRI by the uropathologists and radiologists using a sextant scheme to analyze the correct location of the lesions (12). Details regarding histopathologic matching and lesion annotation are provided in Appendix S1.

Four radiology residents, under the supervision of a board-certified expert radiologist with more than 15 years of experience in prostate imaging, annotated the lesions using ITK-SNAP version 3.80. In all cases, voxel-level MRI-visible suspected lesion annotations were delineated, and patient-level outcomes were recorded.

### Subjective Evaluation of the Rectal Artifacts

To evaluate the diagnostic disparities of TPAS across cases with varying levels of rectal artifacts, we evaluated the rectal artifacts in external testing set 1. We employed a scoring method to assess the impact of rectal artifacts on the prostate gland region. A previous study has demonstrated the good interrater consistency of this method (19). Two radiologists with 4 and 6 years of experience in prostate imaging (L.H. and Z.W., respectively) who were blinded to all clinical details and biopsy results independently performed the rectal artifact subjective evaluation. Discrepancies in scoring between the two radiologists were determined by a senior radiologist with 15 years of experience in prostate imaging (J.Z.). Images were scored using a four-point scale (1: no artifact; 2: mild artifact, when <50% of the peripheral zone next to the rectum was involved; 3: moderate artifact, when 50%–100% of the peripheral zone was affected without involving the transition zone; and 4: severe artifact, when the artifact extended into the transition zone) (19). Examples of common rectal artifacts are shown in Figure S1.

### TPAS-based Model Development

**Figure 2:**
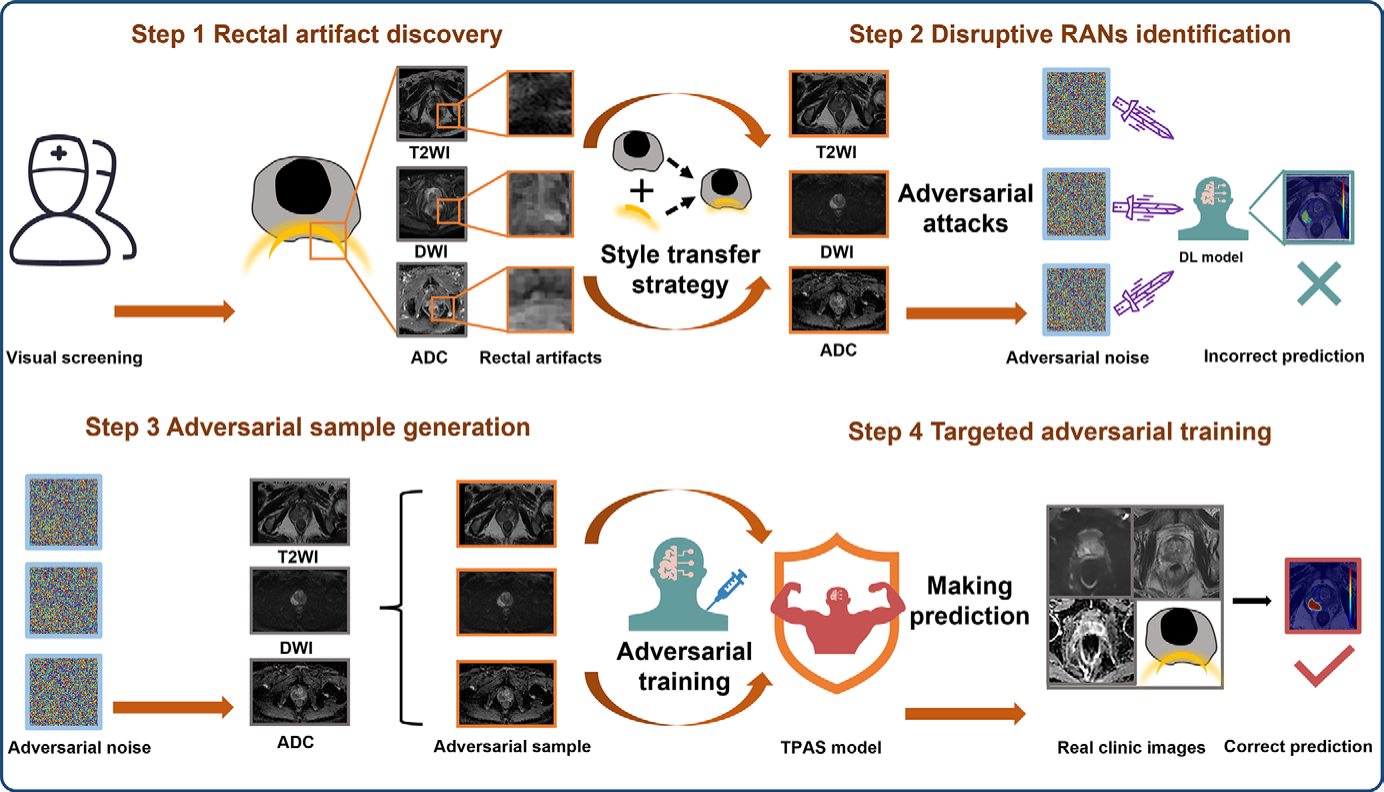
The targeted adversarial training with proprietary adversarial samples (TPAS) strategy diagram. The TPAS strategy includes four steps. Step 1: In the pre-experimental phase, we identified images with severe rectal artifacts that caused misjudgment in multiple DL models. Step 2: We extracted RAN features that disrupted the DL model to generate rectal artifact–pattern adversarial noise. This was achieved by incorporating a style transfer strategy with an adversarial attack strategy. Step 3: We created three types of rectal artifact–pattern adversarial noise, similar to the RAN in T2-weighted imaging, DWI, and ADC studies. These were added to generate proprietary adversarial samples. Step 4: The adversarial samples were then used for targeted adversarial training to enhance the model’s resistance to RAN. ADC = apparent diffusion coefficient, DL = deep learning, DWI = diffusion-weighted imaging, RAN = rectal artifact noise, T2WI = T2-weighted imaging.

As shown in Figure 2, the TPAS-based model development includes four steps:

***Rectal artifact discovery.—*** During the pre-experimental phase, images with severe rectal artifacts that led to misjudgment in multiple DL models were identified.

***Disruptive RAN identification.—*** In this component, we extracted RAN features that disrupted the DL model to generate rectal artifact–pattern adversarial noise by jointly exploiting a style transfer strategy and an adversarial attack strategy. Specifically, to obtain the style features (eg, rectal artifact–related signal intensity variations and morphology) of RAN, a pretrained VGGNet-16 network was used as a feature extractor. This extractor took the style sample as input and grabbed features closely related to the rectal artifact style in its intermediate convolutional layers. The extracted style features were then incorporated into the generated noise on the basis of a style constraint. Meanwhile, to prevent some important semantic contents (eg, lesion structure) of the original samples from being substantially changed during the process of style transfer, we constructed a constraint based on the deeper convolutional layers of the extractor to ensure the consistency of the semantic contents. Furthermore, to motivate the generated noise to have an adversarial ability to interfere with the DL model, we exploited an adversarial attack strategy to optimize the noise to maximize the prediction error of the DL model. More details are provided in Appendix S2.

***Adversarial sample generation.—*** Three types of rectal artifact–pattern adversarial noise, similar to the RAN in T2-weighted imaging, DWI, and ADC studies, were added to generate proprietary adversarial samples (Fig S2). For each type of MR image, an addition operation between the voxel values of the generated noise and those of the original images was performed, followed by a clipping operation to ensure that the upper and lower bounds of the adversarial sample’s voxel values were the same as those of the original image (Fig S3).

**Figure 3:**
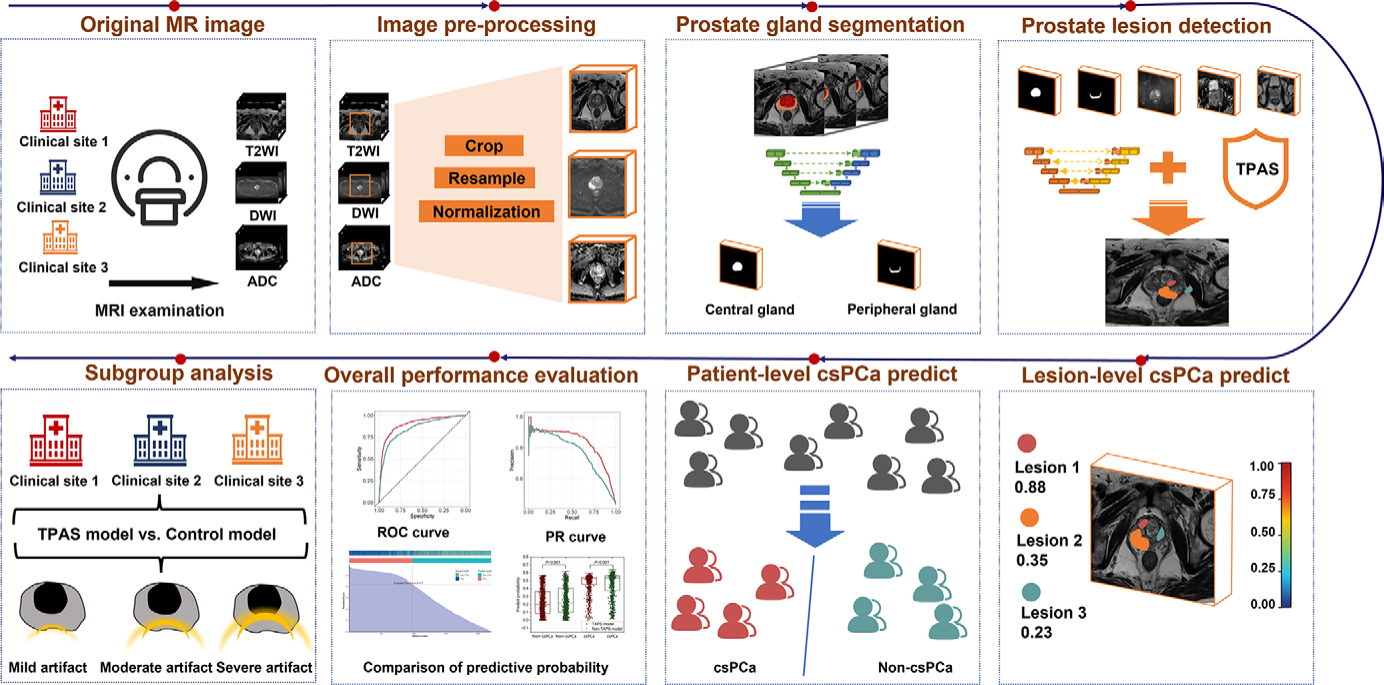
The workflow of the deep learning model for clinically significant prostate cancer (csPCa) detection and diagnosis. The data were collected from three medical centers and preprocessed by cropping, resampling, and normalization. Subsequently, a segmentation model was applied to identify the peripheral and central glands using T2-weighted imaging studies. These segmented regions, along with T2-weighted imaging, DWI, and ADC studies, were input into the diagnostic model. The model generated a lesion confidence map in various prostate regions. Based on this confidence map, corresponding lesion candidates were identified, along with their detection probabilities for csPCa and the patient-based csPCa prediction. Finally, a subgroup analysis was conducted to assess the model’s performance in patients from different medical centers with varying levels of artifacts. ADC = apparent diffusion coefficient, DWI = diffusion-weighted imaging, PR = precision recall, ROC = receiver operating characteristic, TPAS = targeted adversarial training with proprietary adversarial samples, T2WI = T2-weighted imaging.

***Targeted adversarial training.—*** After obtaining adversarial noise, proprietary adversarial samples were added to the model’s training process as additional training data to enhance the model’s resistance to rectal artifacts. During the entire training lifecycle, the generation of the proprietary adversarial samples and optimization of the model parameters are performed alternately; that is, the adversarial samples are continuously updated as the model is improved. The backbone of the model for three-dimensional (3D) csPCa detection and diagnosis (Fig 3) is based on 3D nnU-Net (20). Initially, a prostate gland segmentation model was employed to generate segmentation masks for the central and peripheral glands using T2-weighted images. Subsequently, the prostate lesion detection model used five separate modalities, including segmented central and peripheral gland masks, along with T2-weighted imaging, ADC, and DWI studies as inputs. The model generates a lesion confidence map that indicates the level of confidence that lesions are present in different regions of the prostate. Using the lesion confidence map, corresponding lesion candidates were generated along with their detection probabilities, and the patient-based csPCa prediction score indicated the probability of csPCa. The model was implemented in Python using PyTorch version 1.13.1. Detailed information regarding the data preprocessing, prostate gland segmentation, and lesion detection methods can be found in Appendix S3. The code of the baseline model is available at *github.com/DIAGNijmegen/picai_baseline*.

### TPAS-based Model Evaluation

The model was trained using a fivefold cross-validation approach on the training dataset. The best-performing model, based on its performance during cross-validation, was selected for both internal and external testing. The performance of the TPAS-based model was initially evaluated using the internal testing set and external testing set 1. Subsequently, the impact of imperceptible RAN on the model performance and the resilience of the model to such interference was assessed using external testing sets 2 and 2*. Furthermore, a subgroup analysis was conducted to compare the performance of the model across varying degrees of artifacts and among patients from different medical centers.

### Statistical Analysis

Nonnormally distributed continuous variables were assessed using the Mann-Whitney *U* test. Categorical variables are presented as percentages and were compared using the χ^2^ test. True-positive lesions were predictions that shared a minimum overlap of 0.10 in 3D space with the ground-truth annotation following the challenge and earlier studies (13,21). The interreader agreement of rectal artifact evaluation was assessed using Cohen weighted κ. A linear mixed-effects model was employed to analyze the changes in the csPCa prediction score between the TPAS model and the control model. The fixed effects included the patient group (patients with csPCa vs patients with non-csPCa), model (TPAS model vs control model), clinic sites (Cs 1, Cs 2, and Cs 3), artifact severity (score 1–4), and interactions among these variables. Additionally, a random intercept was used for each patient. The Dice similarity coefficient (DSC) was calculated to evaluate csPCa lesion segmentation performance. The diagnostic performances of the DL model at the patient and lesion levels were evaluated using receiver operating characteristic and precision-recall curves, respectively. Diagnostic performance was compared by calculating the area under the receiver operating characteristic curve (AUC) and the area under the precision-recall curve (AUPRC). The AUC and AUPRC were compared between models using the DeLong test and the bootstrap method, respectively. All statistical analyses were conducted using R statistical software version 3.3.1, and a two-sided *P* value less than .05 was considered statistically significant.

## Results

### Patient Characteristics

A total of 2203 male patients with 3079 MRI-visible lesions from three clinical sites were included in our study for external testing. There were statistically significant differences in age, average prostate-specific antigen levels, PCa incidence rate, and International Society of Urological Pathology grade among the patients from the three centers (*P* < .003). The baseline epidemiologic and clinical characteristics of the patients are shown in Table 1.

**Table 1:**
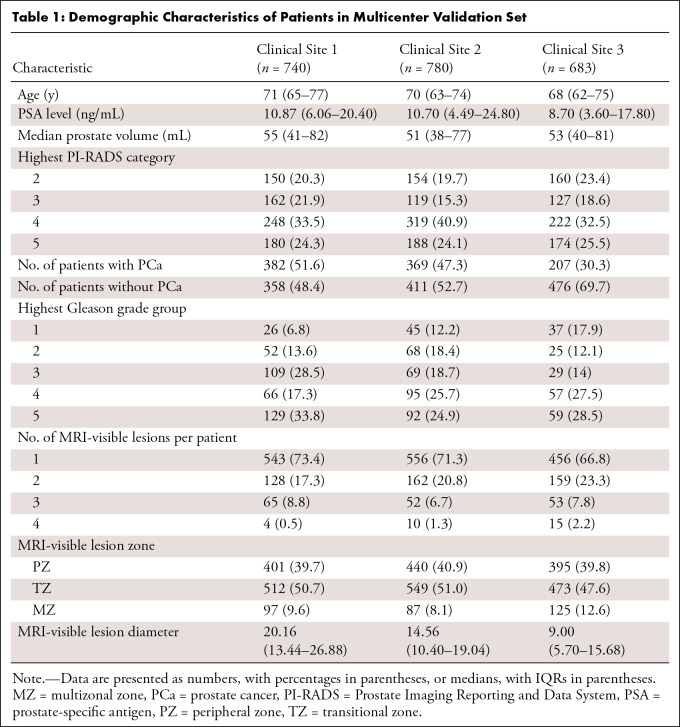
Demographic Characteristics of Patients in Multicenter Validation Set

### Rectal Artifact Evaluation

There was good interreader agreement, with a Cohen weighted κ of 0.77. As shown in Table 2, patients from Cs 1 and Cs 2, who underwent a simple bowel preparation before MRI, had occurrence rates of rectal artifacts that were 6.1% and 12.9% lower, respectively, than patients from Cs 3, who did not undergo any bowel preparation prior to MRI (*P* < .001).

**Table 2:**
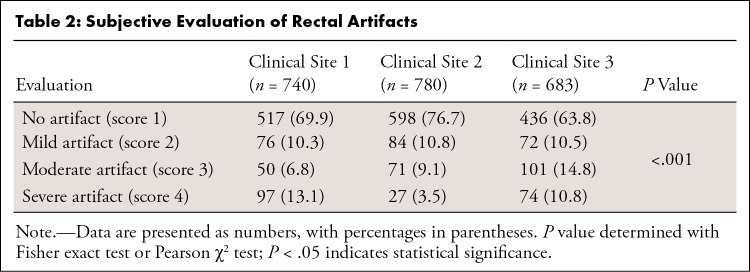
Subjective Evaluation of Rectal Artifacts

### Overall Performance of DL Models with and without TPAS

**Figure 4:**
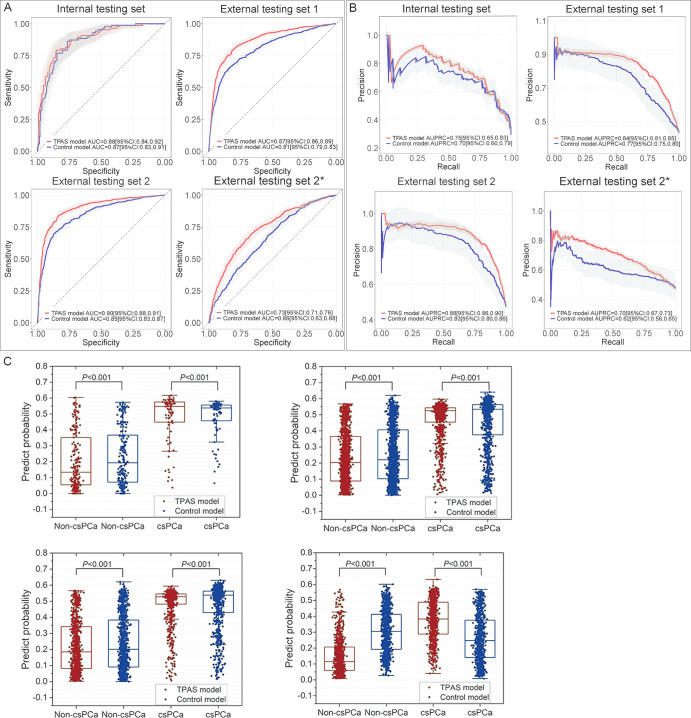
Comparison of the overall performance of deep learning with and without TPAS. **(A)** The receiver operating characteristic curves provide evidence that the TPAS model exhibited superior performance compared with the control model at the patient level in both internal and external testing sets. **(B)** The precision-recall curves indicate that the TPAS model outperformed the control model at the lesion level in both internal and external testing sets. The predictive probability represents the extent to which the model suspects a patient or nodule to be consistent with csPCa. The lightly shaded bands indicate 95% CIs. **(C)** Compared with the control model, the TPAS model yielded a higher mean predictive probability of csPCa lesions and a lower predictive probability of non-csPCa lesions. AUC = area under the receiver operating characteristic curve, AUPRC = area under the precision-recall curve, csPCa = clinically significant prostate cancer, TPAS = targeted adversarial training with proprietary adversarial samples.

**Figure 5:**
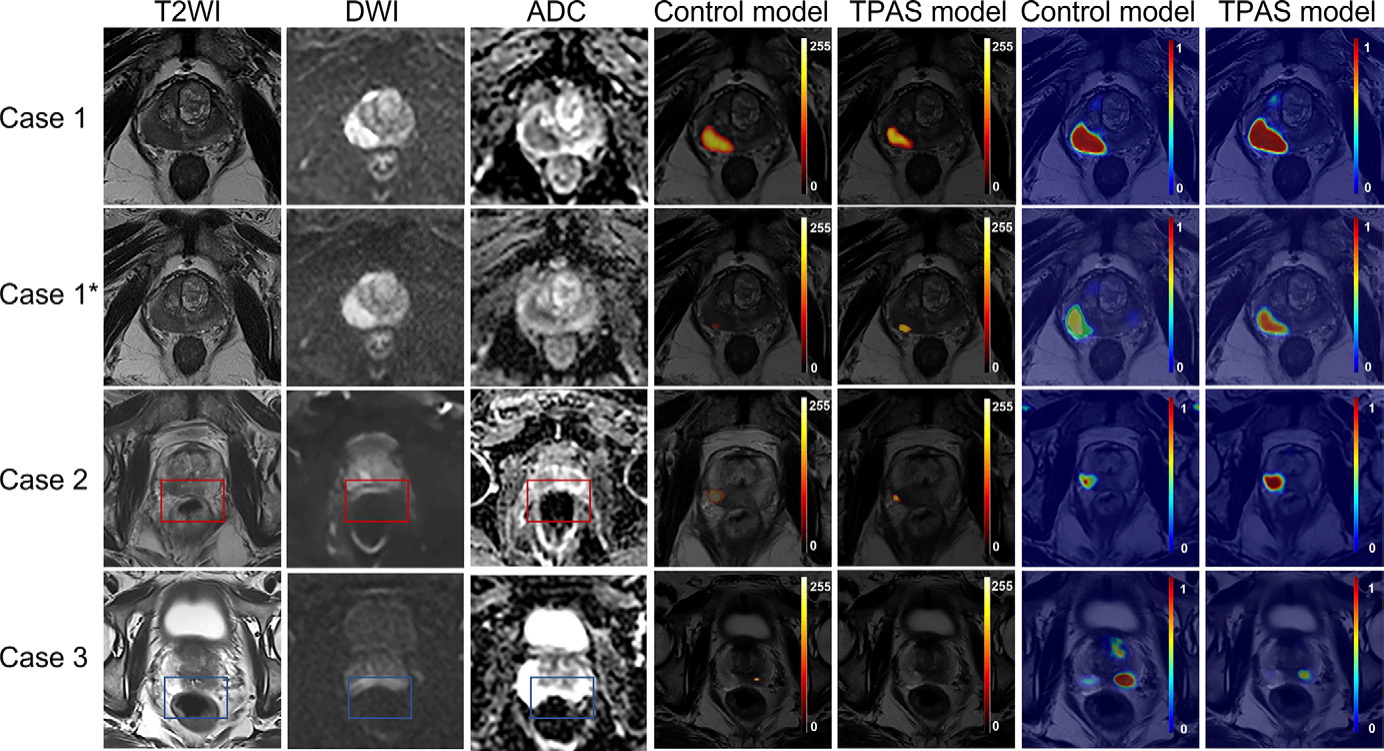
Examples of diagnoses using the deep learning model with and without interference from rectal artifacts. Class activation maps (the fourth and fifth columns from the left) were used to visualize the regions that the model focuses on in an image, whereas lesion confidence maps (the sixth and seventh columns from the left) indicated the detection probabilities of lesion candidates. Case 1: An 85-year-old male patient with PCa in the right PZ (Gleason score 4 + 3). No visible artifacts were observed. The TPAS model and control model both detected the cancer, with probabilities of 0.6 and 0.56, respectively. Case 1*: Rectal artifact–pattern adversarial noise, which is extremely difficult to perceive with the naked eye, was added to case 1 images. The TPAS model still detected the cancer, with a predicted probability of 0.55. However, the control model was affected by the adversarial noise, giving the lesion a predicted probability of only 0.28, which is below the working point (0.45) defined by the receiver operating characteristic curve. Case 2: A 65-year-old male patient with PCa in the PZ (Gleason score 4 + 5). Severe rectal artifacts were present in the images (red rectangles). The TPAS model successfully detected the cancer with a probability of 0.54, whereas the control model missed it with a probability of 0.28. Case 3: A 65-year-old male patient with benign prostatic hyperplasia. Severe rectal artifacts were present in the images (blue rectangles). The TPAS model provided a lesion detection probability of 0.38 in the left PZ, whereas the control model misdiagnosed it as PCa with a detection probability of 0.53. ADC = apparent diffusion coefficient, DWI = diffusion-weighted imaging, PCa = prostate cancer, PZ = peripheral zone, TPAS = targeted adversarial training with proprietary adversarial samples, T2WI = T2-weighted imaging.

As shown in Table 3 and Figure 4, compared with the non-TPAS model, the diagnostic performance of the TPAS model was slightly better in the internal testing dataset but much better in the external testing sets. Both visible rectal artifacts and invisible RAN affected the DL diagnostic performance. Compared with the traditional training method, TPAS reduced the impact of rectal artifacts, allowing the model to focus on true PCa lesions and have better diagnostic performance. Typical examples are shown in Figure 5.

**Table 3:**
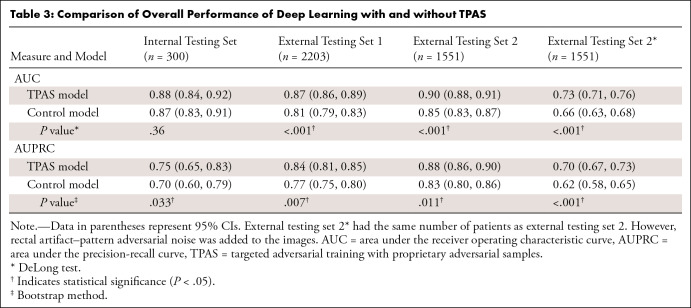
Comparison of Overall Performance of Deep Learning with and without TPAS

The TPAS model exhibited a substantial enhancement in the segmentation performance of csPCa lesions compared with the control model in all testing sets, supported by statistically significant improvements in DSC scores: internal testing set (DSC: 0.66 ± 0.20 [SD] vs 0.50 ± 0.29, *P* < .001), external testing set 1 (DSC: 0.51 ± 0.27 vs 0.38 ± 0.28, *P* < .001), external testing set 2 (DSC: 0.59 ± 0.22 vs 0.45 ± 0.25, *P* < .001), and external testing set 2* (DSC: 0.45 ± 0.19 vs 0.30 ± 0.27, *P* < .001).

Compared with the control model, the TPAS model exhibited a diagnostic performance improvement of 1% at the patient level (AUC: 0.88 [95% CI: 0.84, 0.92] vs 0.87 [95% CI: 0.83, 0.91], *P* = .36) and 5% at the lesion level (AUPRC: 0.75 [95% CI: 0.65, 0.83] vs 0.70 [95% CI: 0.60, 0.79], *P* = .033) in the internal testing set and a diagnostic performance improvement of 6% at the patient level (AUC: 0.87 [95% CI: 0.86, 0.89] vs 0.81 [95% CI: 0.79, 0.83], *P* < .001) and 7% at the lesion level (AUPRC: 0.84 [95% CI: 0.81, 0.85] vs 0.77 [95% CI: 0.75, 0.80], *P* = .007) in external testing set 1. In addition, the TPAS model demonstrated a smaller decrease in the AUC (ΔAUC: −1% vs −6%) and a greater increase in the AUPRC (ΔAUPRC: 9% vs 7%) between the internal testing set and external testing set 1, indicating that the TPAS model has higher diagnostic performance and better stability in real clinical applications than the control model.

The TPAS model also exhibited superior diagnostic performance compared with the control model in external testing dataset 2, which included patients without visible artifacts (AUC: 0.90 [95% CI: 0.88, 0.91] vs 0.85 [95% CI: 0.83, 0.87], *P* < .001; AUPRC: 0.88 [95% CI: 0.86, 0.90] vs 0.83 [95% CI: 0.80, 0.86], *P* = .011). When faced with the addition of rectal artifact–pattern adversarial noise (external testing set 2*), both the models with and without TPAS showed a decrease in diagnostic performance (AUC: 0.73 [95% CI: 0.71, 0.76] vs 0.66 [95% CI: 0.63, 0.68], *P* < .001; AUPRC: 0.70 [95% CI: 0.67, 0.73] vs 0.62 [95% CI: 0.58, 0.65], *P* < .001). However, the TPAS model exhibited a lower degree of performance decline than the control model (ΔAUC: −17% vs −19%, ΔAUPRC: −18% vs −21%; all *P* < .001).

Compared with the control model, the TPAS model shows similar mean predictive probabilities of csPCa (0.48 vs 0.48, *P* = .156) and lower predictive probabilities of non-csPCa (0.15 vs 0.30, *P* < .001) in the internal testing set. Additionally, in the external test datasets, the TPAS model exhibited higher mean predictive probabilities of csPCa lesions (external testing set 1: 0.47 vs 0.45, external testing set 2: 0.48 vs 0.47, external testing set 2*: 0.38 vs 0.26) and lower predictive probabilities of non-csPCa (external testing set 1: 0.23 vs 0.25, external testing set 2: 0.22 vs 0.24, external testing set 2*: 0.15 vs 0.3), with statistically significant differences (all *P* < .001).

### Mixed Linear Model Results

The linear mixed-effects model analysis revealed that the csPCa prediction score was significantly influenced by patient group, clinic site, and artifact severity (patient group: *P* < .001, clinic site: *P* < .001, artifact severity: *P* = .003). Additionally, interactions between model and clinic site (*P* < .001), clinic site and artifact severity (*P* = .002), as well as the three-way interaction between model, clinic site, and patient group (*P* < .001) and model, clinic site, and artifact severity (*P* < .001) were also found to be significantly related to predicting the csPCa prediction score. (More details are shown in Table S3.)

### Subgroup Analysis of DL with and without TPAS

**Figure 6:**
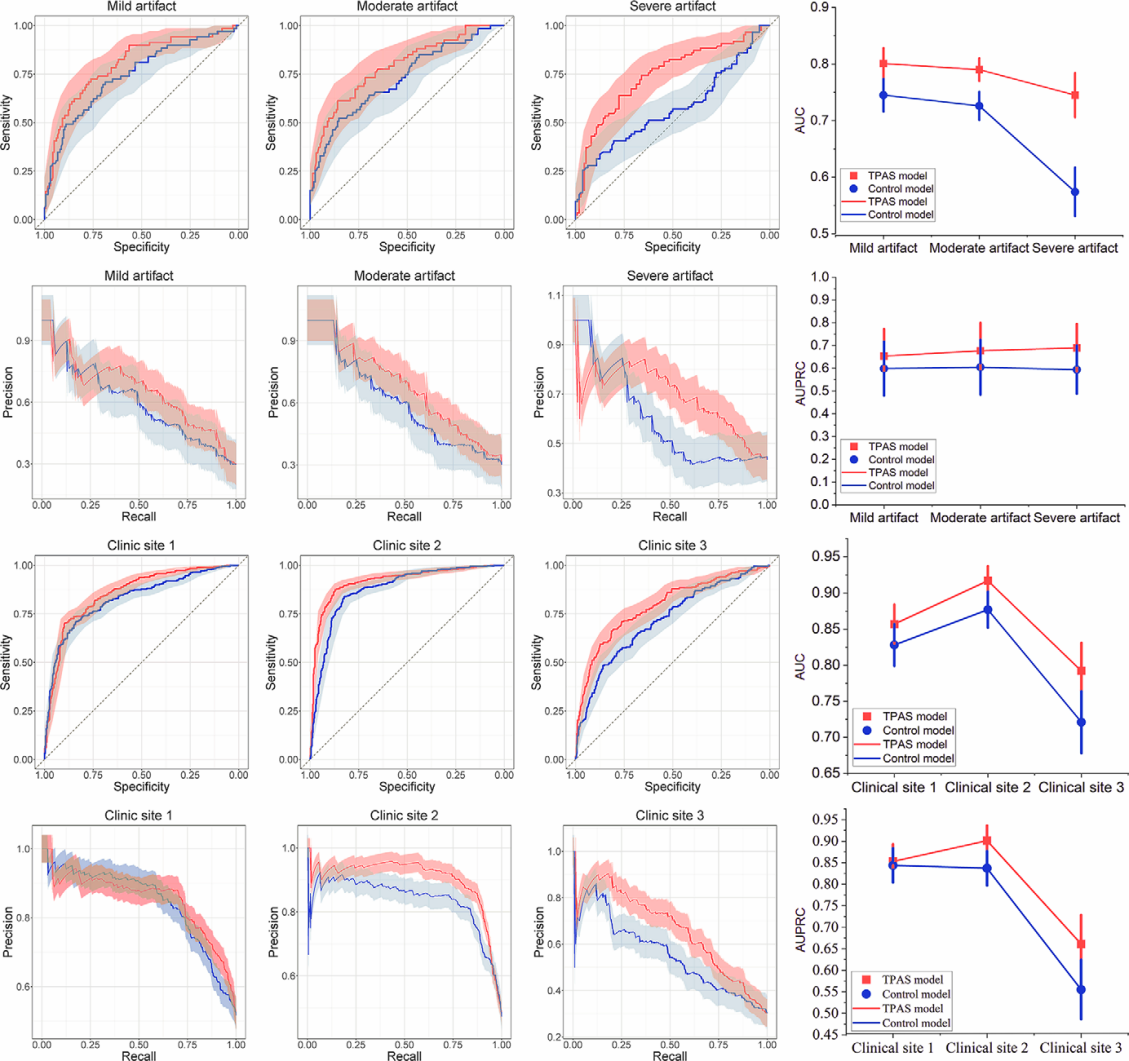
Subgroup analysis of deep learning with and without TPAS. **(A)** The receiver operating characteristic (ROC) and **(B)** precision-recall (PR) curves from the subgroup analysis concerning the severity of rectal artifacts demonstrate that the TPAS model outperformed the control model on datasets with varying degrees of artifacts. Furthermore, as the artifacts become more severe, the diagnostic advantage of the TPAS model increases. **(C)** The ROC and **(D)** PR curves from the subcomponent analysis concerning different medical centers show that the TPAS model performed better than the control model at different medical centers. Moreover, in a dataset with a high occurrence rate and severe rectal artifacts (clinic site 3), the diagnostic advantage of the TPAS model becomes more pronounced. AUC = area under the ROC curve, AUPRC = area under the PR curve, TPAS = targeted adversarial training with proprietary adversarial samples.

As shown in Table 4 and Figure 6, the TPAS model performed better than the control model in patients with rectal artifacts. The more severe the artifacts, the greater the advantage of TPAS (mild artifacts: AUC: 0.80 [95% CI: 0.74, 0.87] vs 0.75 [95% CI: 0.67, 0.82], AUPRC: 0.65 [95% CI: 0.54, 0.75] vs 0.60 [95% CI: 0.48, 0.71]; moderate artifacts: AUC: 0.79 [95% CI: 0.72, 0.86] vs 0.73 [95% CI: 0.65, 0.80], AUPRC: 0.68 [95% CI: 0.56, 0.78] vs 0.61 [95% CI: 0.48, 0.71]; and severe artifacts: AUC: 0.75 [95% CI: 0.67, 0.82] vs 0.57 [95% CI: 0.49, 0.66], AUPRC: 0.69 [95% CI: 0.59, 0.78] vs 0.59 [95% CI: 0.49, 0.69]). There was no evidence of a difference in the AUC between the two models in patients with mild artifacts (*P* = .08), but the differences were significant when assessing patients with moderate and severe artifacts (all *P* < .02).

**Table 4:**
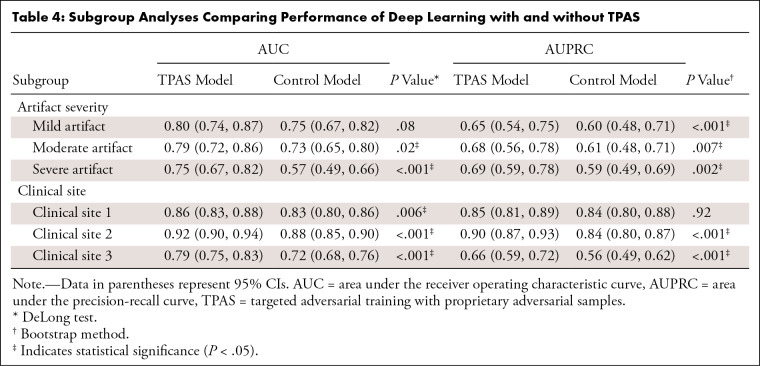
Subgroup Analyses Comparing Performance of Deep Learning with and without TPAS

The TPAS model also performed better than the control model at all clinical sites. Both models performed better in patients from Cs 1 (TPAS vs control: AUC: 0.86 [95% CI: 0.83, 0.88] vs 0.83 [95% CI: 0.80, 0.86]; AUPRC: 0.85 [95% CI: 0.81, 0.89] vs 0.84 [95% CI: 0.80, 0.88]) and Cs 2 (TPAS vs control: AUC: 0.92 [95% CI: 0.90, 0.94] vs 0.88 [95% CI: 0.85, 0.90]; AUPRC: 0.90 [95% CI: 0.87, 0.93] vs 0.84 [95% CI: 0.80, 0.87]) than in patients from Cs 3 (TPAS vs control: AUC: 0.79 [95% CI: 0.75, 0.83] vs 0.72 [95% CI: 0.68, 0.76]; AUPRC: 0.66 [95% CI: 0.59, 0.72] vs 0.56 [95% CI: 0.49, 0.62]). There was no evidence of a difference in the AUPRC between the two models for patients from Cs 1 (*P* = .915), but the differences between the models in the AUC for patients from Cs 1 and in the AUC and AUPRC for patients from Cs 2 and Cs 3 were all significant (all *P* < .006).

## Discussion

Our proposed training with a proprietary adversarial sample model enhanced the ability of DL models to adapt to the interference of rectal artifacts, thus improving the diagnostic accuracy and stability of the model in cases with rectal artifacts. This study provides valuable insights for further advancements in DL model training strategies for PCa diagnosis.

Although previous research has highlighted the efficacy of adversarial training in bolstering deep neural networks against adversarial noises for tasks like natural image segmentation (22) or detection (23), adversarial noise for medical images (eg, MRI data) has not received sufficient attention because of the methods of producing medical images and the substantial differences between medical image noise and natural noise (24,25). A previous tentative study indicated that adversarial training could enhance the robustness and generalizability of models for csPCa, leading to improved diagnostic accuracy across varied testing datasets (26). Notably, this previous study was confined to examining the role of adversarial training in two-dimensional DL models for csPCa classification, without extending its evaluation to 3D DL models or diverse tasks like csPCa detection and segmentation. Moreover, the general adversarial training employed in this prior study was designed for natural images, failing to replicate the distinctive noise characteristics of MR images, specifically rectal artifacts that arise during MRI scans. Consequently, the general adversarial training exhibited limited efficacy in handling the unique and disruptive RAN. In our study, we sought to address these limitations by integrating clinical prior knowledge into the generation of adversarial noise. This approach aimed to identify noisy characteristics from rectal artifacts that could interfere with the DL model’s judgments and then model these features to create specific adversarial samples. By utilizing knowledge transfer and adversarial attack techniques, we developed specialized adversarial samples incorporating rectal artifact–pattern adversarial noise. These samples were then leveraged to optimize the parameters of our automated 3D model for csPCa diagnosis. Our findings demonstrated improved performance compared with the aforementioned study.

In our study, the TPAS model achieved similar or better performance than most DL models published in previous studies (12,27–33), which mainly focused on improving model diagnostic performance by modifying the DL structure or increasing the sample size of the training dataset (21). A systematic review of state-of-the-art DL models for the diagnosis and localization of csPCa using MRI between 2019 and 2022 (34) reported an average AUC of 0.82 (range: 0.76–0.86) for DL systems in diagnosing csPCa at the patient level. With additional training data, two studies published in 2023 reported slightly better results. Sun et al (35) proposed a cascade 3D U-Net–based model to detect and localize visible csPCa, demonstrating a patient-level AUC of 0.88. Karagoz et al (21) trained a self-adapting 3D nnU-Net model with an AUC of 0.89 using the Prostate Imaging: Cancer AI Challenge data (13). However, these models exclude cases with poor image quality or severe artifacts. This results in the possibility of overestimating diagnostic performance, and the performance of these models on a dataset with rectal artifacts remains unclear. In contrast to these studies, no images were excluded in our study because of the presence of rectal artifacts, even severe artifacts. Therefore, the external testing results of our study are closer to those of real clinical scenarios.

Our results indicate that the presence of rectal artifacts can affect the diagnostic performance of the model, which is consistent with the results of a previous study (2). In this previous study, rectal artifacts on DW images were found to be independent risk factors leading to misdiagnosis when using a commercial DL system (MR Prostate AI v1.2.5; Siemens Healthcare) (2). However, that study did not examine the specific impact of rectal artifacts on DL performance. Here, the control model had poorer performance than the TPAS model as the severity of the artifacts increased. In addition, even subtle invisible rectal artifact–pattern adversarial noise can mislead DL models, resulting in a decrease in diagnostic performance. Therefore, we can infer that taking measures to reduce visually perceptible artifacts at the image scanning level and using TPAS during model training to enhance the model’s resistance to subtle imperceptible RAN can effectively reduce the interference of rectal artifacts, resulting in better diagnostic performance and stability in real clinical applications.

The linear mixed-effects model results underscore the substantial impact of clinic sites and artifact severity and interactions between these two factors on predicting the csPCa prediction score. We compared model performance across three different medical centers and found that the DL models performed better in centers that implemented bowel preparation before MRI than in the center without any bowel preparation, indicating that simple bowel preparation, such as a fluid diet and emptying the rectum prior to MRI, is also a useful strategy to enhance the model’s diagnostic performance. However, considering the conflicting evidence regarding the effectiveness of certain bowel preparation strategies (such as enemas, dietary modifications, and antiperistaltic agents) on prostate MR image quality (8), the effectiveness of other bowel preparation strategies remains uncertain and requires further research and evidence-based investigation.

Our study had several limitations. First, we relied on biopsies as reference standards, and using whole-mount histopathology could potentially enhance the accuracy of the agreement between MRI and histopathology results. Second, our training and evaluation of DL models were exclusively focused on MRI-visible csPCa, implying the possibility of DL models missing MRI-invisible lesions. Additionally, our investigation addressed the impact of only rectal artifacts on DL models. Nevertheless, in clinical practice, various artifacts can occur in prostate MRI, and it remains uncertain whether these artifacts might interfere with the model’s diagnosis. To address these limitations, our future research will explore the potential effects of other artifacts on DL models and develop corresponding training strategies to mitigate these potential risks, ultimately aiming to enhance the accuracy and stability of the model in real clinical scenarios.

In conclusion, reducing rectal artifact interference can significantly enhance the effectiveness of DL for MRI-based PCa diagnosis in real clinical applications. The TPAS-based model, which addresses the challenges posed by rectal artifacts, shows promising potential as a valuable tool for accurate and reliable PCa diagnosis. Implementing this model in clinical practice could lead to improved diagnostic outcomes and better patient care in the context of PCa assessment.
